# Neurological signs as early determinants of dementia and predictors of mortality among older adults in Latin America: a 10/66 study using the NEUROEX assessment

**DOI:** 10.1186/s12883-018-1167-4

**Published:** 2018-10-03

**Authors:** Lorenzo Pasquini, Jorge Llibre Guerra, Martin Prince, Kia-Chong Chua, A. Matthew Prina

**Affiliations:** 1 Memory and Aging Center, Department of Neurology, University of California San Francisco, 675 Nelson Rising Lane, San Francisco, CA 94143 USA; 2 Global Brain Health Institute, Memory and Aging Center, University of California San Francisco, 675 Nelson Rising Lane, San Francisco, CA 94143 USA; 3 Neurology and Neurosurgery Institute, 139 Calle 29, 10400 Havana, Cuba; 4 King’s College London, Health Service and Population Research Department, Centre for Global Mental Health, Institute of Psychiatry, Psychology & Neuroscience, De Crespigny Park, London, SE5 8AF UK

**Keywords:** Dementia, Epidemiology, Low and middle-income countries, Mortality, Neurological signs

## Abstract

**Background:**

Neurodegenerative processes in the elderly damage the brain, leading to progressive, incapacitating cognitive, behavioral, and motor dysfunctions which culminate in dementia. Fully manifest dementia is likely to be preceded by the presence of neurological signs, which could serve as early determinants of dementia and predictors of mortality. The aims of this study were to assess the construct validity of a neurological battery assessed among older adults living in Latin America, and to test the association of groups of neurological signs with dementia cross-sectionally, and mortality longitudinally.

**Methods:**

The 10/66 Dementia Research Group collected information on neurological symptoms via the NEUROEX assessment in population based surveys of older adults living in low and middle-income countries. Data from 10,856 adults participating in the baseline assessment of the 10/66 study and living in Cuba, Dominican Republic, Peru, Venezuela and Mexico were analysed. Exploratory and confirmatory analysis were used to explore dimensionality of neurological symptoms. Poisson regression analyses were used to link groups of neurological signs with dementia at baseline. Cox hazard regression models were used to explore the predictive validity of neurological signs with mortality at follow up.

**Results:**

Exploratory and confirmatory factor analyses revealed four dimensions of neurological signs, which are associated with lesions of specific brain regions. The identified factors showed consistency with groups of neurological signs such as frontal, cerebellar, extrapyramidal, and more generalized gait disturbance signs. Regression analyses revealed that all groups of neurological signs were positively associated with dementia at baseline and predicted mortality at follow up.

**Conclusions:**

Our findings support the construct and predictive validity of the NEUROEX assessment, linking neurological and gait impairments with dementia at baseline, and with mortality at follow up among older adults living in five Latin American countries.

**Electronic supplementary material:**

The online version of this article (10.1186/s12883-018-1167-4) contains supplementary material, which is available to authorized users.

## Background

As demographic ageing advances, many low and middle-income countries (LMICs) are experiencing a health transition, where non-communicable diseases assume a progressively greater significance. Non-communicable diseases are already the leading cause of death in all world regions apart from sub-Saharan Africa [1]. Given the dynamic interaction of mental health and physical illnesses, neurological and mental disorders need particular attention [2]. Among all mental health disorders, dementia accounts for a large proportion of mortality and years lived-with-disability targeting older adults in LMICs [3, 4]. In neurological disorders such as dementia, early neurological signs are also common in advanced age [5, 6], and are likely to be the result of the ongoing neurodegenerative brain processes taking place several years before first cognitive symptoms appear [7, 8]. Distinct neurological signs are typically anchored on distinct brain regions, and relate to lesions of specific brain structures [9–11]. For example, sequencing tasks and frontal “release” signs are commonly associated with lesions in the frontal lobes as found in frontotemporal and vascular dementia [10, 12]. The inability to perform fast alternating movements – e.g. dysdiadochinesia – is commonly related to lesions of the cerebellum, while symptoms such as tremor and rigidity are indicators of extrapyramidal lesions in the striatopallidonigral system as found in Parkinson’s disease [13]. Finally, diffuse disorders of motor function may contribute to impairments of stance and gait.

Notably, a consistent link between several neurological signs, dementia and mortality has been reported in several studies from high income countries [14–19]. Such findings indicate a potential use of neurological symptoms as early determinants of dementia and predictors of mortality among older adults. This may be particular relevant to LMICs, where the older population may experience limited access to advanced diagnostic tools of dementia such as cerebrospinal fluid and neuroimaging biomarkers [7].

The 10/66 Dementia Research Group, a collective of researchers carrying out population-based research into dementia, non-communicable diseases and ageing in LMICs, has started tackling this issue by carrying out population-based surveys using standardised methodology across a large number of LMICs (Cuba, China, Dominican Republic, India, Mexico, Nigeria, Peru, Puerto Rico, & Venezuela). 10/66 refers to the fact that when the group was created in the late 90s, two-thirds (66%) of people with dementia were living in LMICs, and that 10% or less of population-based research had been carried out in those regions. The 10/66 Dementia Research Group included an assessment of neurological symptoms (NEUROEX) as part of population-based surveys investigating dementia and ageing in LMICs [20, 21]. In order to generate evidence about the construct and predictive validity of the NEUROEX assessment, the purposes of this study are to *(i)* explore the dimensionality of neurological symptoms and their link to groups of neurological signs; *(ii*) provide evidence for a positive association relating groups of neurological signs with dementia at baseline, and mortality at follow up in older adults living in five Latin American countries.

## Methods

### Design

Secondary analyses were performed on data from the 10/66 Dementia Research Group surveys of representative samples of older people living in five Latin American countries (urban sites in Cuba, Dominican Republic and Venezuela, and rural and urban sites in Mexico and Peru). Full details of the study protocol can be found elsewhere [20, 21]. Briefly, a cross-sectional one phase survey was carried out in geographically defined catchment areas. All residents aged 65 years and over were included in the survey and an informant was also interviewed. The sample size for each country was between 2000 and 3000 participants. All participants underwent a comprehensive interview, including a structured clinical interview, a physical examination, an assessment of neurological symptoms and an informant interview. The interviews generally took place at the participant’s home and were translated in Spanish. Vital status was determined 3–5 years after baseline survey. A detailed account reporting mortality assessment, causes and rates has been provided in previous studies [22]. All studies were approved by local ethical committees and by the King’s College London ethical committee.

### Measurements

#### 10/66 dementia

Dementia was ascertained according to the Diagnostic and Statistical Manual of Mental Disorders IV (DSM-IV) [23] and the cross-culturally validated 10/66 dementia diagnosis algorithm [24]. For 10/66 dementia diagnosis, a logistic regression model was used to calculate coefficients linked to outputs from a structured clinical mental-state interview [25]. The battery included: a) the Geriatric Mental State [26], b) two cognitive tests; the Community Screening Instrument for Dementia (CSI’D’) COGSCORE [27] and the modified CERAD 10 word list learning task with delayed recall [28], and c) informant reports of cognitive and functional decline from the CSI’D’ RELSCORE [27]. 10/66 dementia diagnosis has been shown to be highly sensitive and specific and was given to participants scoring above a cut-point of predicted probability for dementia derived from the aforementioned calculated coefficients [24].

#### Socio-demographical variables and general health indicators

Beside the NEUROEX assessment, socio-demographical and general health indicators were assessed for each country by the interviewers asking participants or a key informant. Socio-demographical variables included gender, educational level, food insecurity (yes no; assessed by the interviewer by asking “have you ever gone hungry because of lack of food?”), income (yes or no; combined measure derived by the interviewer’s question asking whether participants received any income, any pension or proxy measure of household income), number of assets and age. General health indicators were assessed by the interviewer if not specified differently and included depression (EURO-D depression scale) [29], care dependence (based on open-ended questions administered by the interviewer to the key informant) [30], having had a stroke diagnosed (yes or no), having had a diagnosis of diabetes (yes or no), hypertension (blood pressure > =140/90 or antihypertensive treatment, assessed by the interviewer) and dementia (yes or no; based on the 10/66 dementia algorithm) [24].

#### NEUROEX assessment

The NEUROEX assessment was conducted by local trained health workers and health professionals and generally took place within participants’ homes and included a brief fully structured neurological assessment with quantifiable measures of lateralising signs, parkinsonism, ataxia, apraxia and primitive “release” reflexes [20] (see Appendix in the Additional file 1 for more details). Based on data completeness, the following NEUROEX items were included in the analyses: vertical gaze; glabellar and pout reflexes; tremor; rigidity; cogwheeling; fist-palm-side sequencing and reciprocal coordination sequencing; fine finger movement; dysdiadochokinesis (speed and coordination); ataxia; bradykinesia; bilateral armswing; gait steps and time needed to walk five metres. Only the presence of bilateral impairments was considered as pathological, since our main interest was to test the link between neurological symptoms and fully manifest dementia or mortality, rather than explore the association to incipient dementia. Bilateral items where dichotomised and testlets were then created to combine these highly correlated bilateral items, which would have likely created multicollinearity issues in further analyses. The only exceptions were for tremor, where items where summed after dichotomisation, and rigidity and cogwheeling, where items where just summed without dichotomisation.

### Statistical analyses

All statistical analyses were performed in STATA v.12. We estimated the prevalence of neurological symptoms based on the testlets derived from the NEUROEX assessment battery for each country and across sites. For the only continuous NEUROEX variables used in the analysis, gait steps and time, the median (Q2) and interquartile range (Q1-Q3) were reported.

#### Exploratory and confirmatory factor analysis

In order to avoid circular analysis, the whole dataset was randomly divided in two samples, a smaller one containing 30% of the data, and a bigger one containing 70% of the data. Exploratory factor analysis was used to estimate dimensionality of the selected NEUROEX items on the smaller containing 30% of the data. A four-factor solution principal component analysis was performed on the selected NEUROEX items based on the correlation matrix [31, 32]. Bartlett’s test of sphericity, the Kaiser-Meyer-Olkin (KMO) measure of sampling adequacy, Kaiser’s criterion, scree tests and Horn’s parallel analysis were used as additional criteria to estimate exploratory factor analysis reliability. The cut off used for item loading on a given factor was 0.3. A varimax rotation was carried out and an eigenvalue of one was chosen as initial extraction criterion [33] (see Additional file 1: Supplementary methods).

Based on the output of the exploratory factor analysis across sites, we subsequently tested and compared the goodness-of-fit of a four-factor solution model between sites using confirmatory factor analysis on the second sample containing 70% of the data [33]. All further analyses were performed on this sample. Absolute and relative indices were used to test goodness-of-fit: the Akaike’s Information Criterion (AIC) – the lower the AIC value, the better the fit of the model [34]; the Tucker–Lewis Index (TLI) [35] – values greater than 0.80 are considered acceptable; and the root mean square error of approximation (RMSEA) [36] – values between 0.05 to 0.08 indicate reasonable fit for the model.

#### Factor scores

Individual factor scores were derived from the confirmatory factor analysis across sites via regression analysis. Independent variables in the regression equation were the standardised observed values of the items in the estimated factors. These predictor variables were weighted by regression coefficients, obtained by multiplying the inverse of the observed variable correlation matrix by the matrix of factor loadings. The factor scores were the dependent variables in the regression equation. The computed factor scores were subsequently standardised to a mean of zero with a standard deviation of 1.0 [37]. Factor scores were finally trichotomised based on the empirical distribution of the continuous regression, with half of the standard deviation of 1.0 (0.5 z-value) used as threshold to define three categories reflecting a clinical continuum of respectively neurologically less-impaired (z-values ≤0.0; coded as 0), mildly-impaired (0.0 < z-values ≥0.5; coded as 1) and severely-impaired (0.5 < z-values; coded as 2) subjects. Because of the unusual distribution of factor scores belonging to the latent factor later identified as gait disturbance signs, we carried out additional sensitivity analyses where data binning procedures were applied on these factor scores. One categorisation was based on the 90th percentile resulting in two categories of less-impaired (z-values ≤90th percentile) and impaired (z-values >90th percentile) subjects. A second binning was applied by dividing the factors scores in five bins by steps of 2 z-values resulting in five categories of less-impaired (z-values ≤0.0), mildly-impaired (0.0 < z-values ≥2.0), moderately-impaired (2.0 < z-values ≥4.0), heavily-impaired (4.0 < z-values ≥6.0) and severely-impaired (6.0 < z-values) subjects.

#### Regression analyses

Poisson regression analyses were used to investigate the relationship between 10/66 dementia and neurological domains. For each latent variable, three types of models were run: a) an unadjusted model with only the factor scores as categorical variables; b) a second model correcting for socio-demographical variables including gender, educational level, food insecurity, income insecurity, number of assets and age as additional covariates in each model; c) a final fully adjusted model including the former variables and general indicators of health status such as depression (EURO-D depression scale), care dependence, clinically diagnosed stroke, diabetes and hypertension.

Finally, Cox proportional hazard regression analyses were run to predict mortality at follow up from the factor scores [22, 38]. Identical adjustments as in the Poisson regression analysis were carried out. The final models were also adjusted for 10/66 dementia. For the later identified gait disturbance signs, additional sensitivity analyses were carried out by using alternative data binning procedures as explained above. All regression analyses were performed on the dataset across sites.

## Results

Socio-demographic characteristics (Table 1) were comparable across countries. Participants tended to be younger in the sample from Venezuela, and to have fewer years of education when coming from Mexico and the Dominican Republic. The number of assets was lower in the Dominican Republic, where participants also experienced the highest food and income insecurity. Participants from Cuba experienced the lowest food and income insecurity. At baseline, 9.5% of participants were diagnosed with 10/66 dementia; 17.0% of participant assessed at baseline died before the follow-up interview.

**Table 1:** Socio-demographic characteristics by individual countries and over pooled countries

Absolute values (%)	Across sites	Cuba	Dominican Republic	Peru	Venezuela	Mexico
Number of subjects	10,856 (100.0)	2941 (27.1)	2000 (18.5)	1931 (17.8)	1534 (18.1)	2002 (27.1)
Age						
65–69	3230 (29.8)	760 (25.9)	533 (26.4)	554 (28.6)	839 (41.8)	544 (27.1)
70–74	2852 (26.3)	789 (26.9)	520 (25.9)	493 (25.5)	469 (23.6)	581 (29.0)
75–79	2206 (20.3)	639 (21.7)	397 (19.7)	399 (207)	345 (18.4)	426 (21.3)
> 79	2555 (23. 6)	749 (25.5)	561 (27.9)	486 (25.2)	308 (16.3)	451 (22.5)
Female	6941 (62.9)	1913 (65.0)	1325 (65.9)	1183 (61.2)	1252 (64.6)	1268 (63.3)
Educational level						
None	1298 (12.0)	75 (2.5)	392 (19.7)	121 (6.3)	156 (8.1)	554 (27.7)
Some, did not complete primary	3217 (29.9)	655 (22.3)	1022 (51.3)	231 (12.0)	445 (23.1)	864 (43.2)
Completed primary	3392 (31.5)	979 (33.3)	370 (18.6)	727 (37.9)	965 (50.1)	351 (17.5)
Completed secondary	1770 (16.4)	728 (24.8)	135 (6.8)	517 (27.0)	266 (13.8)	124 (6.2)
Tertiary	1094 (10.2)	499 (17.0)	73 (3.7)	321 (16.7)	93 (4.8)	108 (5.4)
Number of assets						
0–3 assets	1673 (15.4)	451 (15.4)	643 (32.0)	155 (8.0)	48 (2.4)	376 (18.8)
4–5 assets	4596 (42.4)	876 (29.8)	444 (22.1)	1134 (58.7)	0 (0.0)	844 (42.1)
6 assets	2152 (19.8)	1073 (36.5)	733 (36.5)	181 (9.4)	1298 (66.1)	165 (8.2)
More than 6 assets	2422 (22.3)	536 (18.3)	186 (9.3)	463 (23.9)	619 (31.5)	618 (30.8)
Food insecurity	752 (7.0)	140 (4.8)	240 (12.1)	137 (7.2)	111 (6.0)	124 (6.2)
Income insecurity	4433 (40.8)	527 (17.9)	1400 (69.6)	668 (34.6)	818 (41.6)	1020 (50.9)

### Prevalence and distribution of neurological symptoms

There was considerable variation in the prevalence and distribution of neurological symptoms (Table 2). Overall, Cuba had the lowest prevalence of neurological symptoms while the Dominican Republic had the highest. For instance, the prevalence of decreased armswing and bradykinesia was respectively of 14.8% and 13.0% in Cuba and 36.8% and 33.4% in the Dominican Republic. The most common neurological symptoms across sites were the glabellar reflex (29.0%) and first palm side sequencing (34.4%), whereas the least common were cogwheeling (9.9%), dysdiadochokinesia speed (9.3%) and coordination (8.7%).

**Table 2:** Prevalence of neurological symptoms derived from the NEUROEX assessment by individual country and for pooled countries

NEUROEX Absolute numbers (%)	Across sites	Cuba	Dominican Republic	Peru	Venezuela	Mexico
Glabellar reflex more than 4 taps	3011 (29.0)	1095 (37.3)	367 (18.4)	252 (13.1)	536 (35.2)	761 (38.3)
Pout reflex present	1575 (15.2)	244 (8.0)	382 (19.2)	95 (4.9)	181 (11.9)	673 (33.6)
FPS sequencing unsuccessful after 5 demonstration	3513 (34.4)	750 (26.0)	823 (42.0)	277 (14.5)	431 (28.7)	1232 (62.8)
Reciprocal sequencing unsuccessful after 5 tries	2911 (28.2)	796 (27.2)	385 (19.6)	229 (11.9)	442 (29.6)	1059 (52.9)
Tremor at least one limb	1348 (13.0)	222 (7.8)	224 (11.2)	213 (11.0)	264 (17.4)	425 (21.2)
Cogwheeling at least one limb	832 (9.9)	183 (6.2)	96 (4.8)	221 (11.5)	106 (7.2)	226 (11.3)
Rigidity at least one limb	1549 (15.0)	374 (12.7)	272 (13.7)	278 (14.5)	291 (19.6)	334 (16.7)
Fine finger movement	1214 (11.7)	207 (7.1)	236 (12.0)	184 (9.6)	221 (14.8)	336 (18.3)
Dysdiadochokinesia Speed	970 (9.3)	242 (8.2)	160 (8.1)	231 (12.0)	99 (6.6)	238 (12.0)
Dysdiadochokinesia coordination	910 (8.7)	187 (6.4)	143 (7.3)	168 (8.7)	90 (6.0)	322 (16.1)
Armswing	2160 (21.2)	432 (14.8)	702 (36.8)	276 (14.3)	215 (15.3)	535 (26.7)
Ataxia	1413 (13.9)	234 (8.0)	428 (22.4)	266 (13.8)	125 (8.8)	360 (18.0)
Bradykinesia	1949 (19.1)	382 (13.0)	637 (33.4)	328 (17.0)	178 (12.6)	424 (21.2)
Gait steps Q2 (Q1-Q3) in steps	18 (14–22)	20 (17–24)	20 (18–24)	18 (15–20)	17 (11–20)	12 (10–15)
Gait time Q2 (Q1-Q3) in seconds	14 (10–18)	15 (12–20)	17 (15–23)	14 (10–18)	12 (9–15)	8 (7–11)

*FPS* fist palm side

### Exploratory and confirmatory factor analysis

**Fig. 1:**
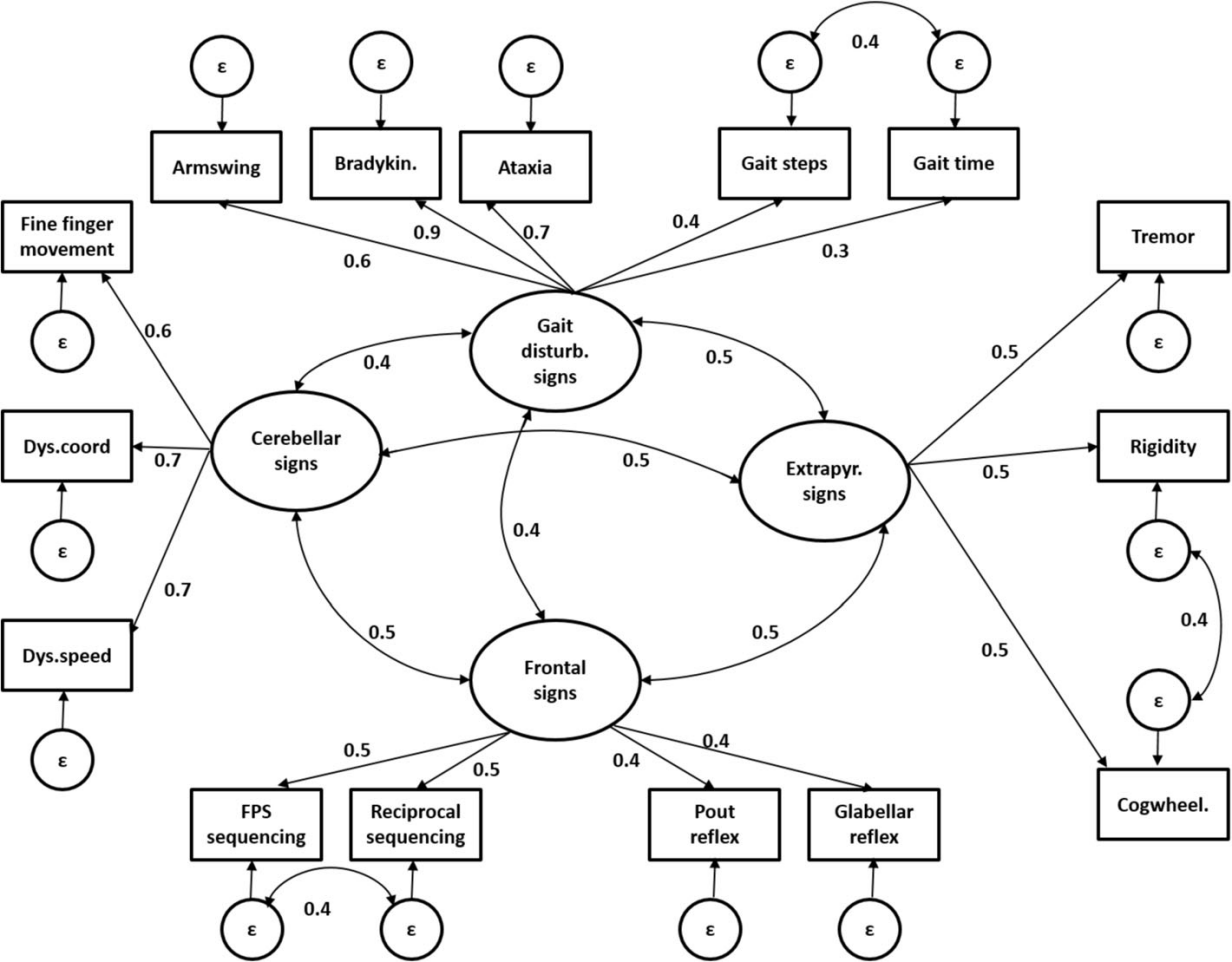
Schematic representation of the model used for confirmatory factor analysis with corresponding loading coefficients from the analysis pooled across sites. Rectangles reflect observed variables; ovals reflect latent variables and circles error terms of the model (ε). Simple arrows and corresponding values reflect the factor paths and loadings of observed variables to the latent variables and of the error terms to the corresponding observed variable; double arrows and corresponding values reflect covariance between latent variables or error terms. Bradykin. = Bradykinesia; Cogwheel. = Cogwheeling; Dys.coord = Dysdiadochokinesia coordination; Dys.speed = Dysdiadochokinesia speed; ε = Error term; FPS = Fist palm side; Extrapyr. = Extrapyramidal; Gait disturb. = Gait disturbance

Exploratory factor analysis was performed through a four-factor principal component analysis on randomly selected 30% of the data (see Additional file 1: Table S1). Qualitative and statistical criteria confirmed analysis suitability. All factors together explained 51.0% of the total variance. The loading structure of the dataset across sites classified vertical gaze as not consistently loading on any factor (factor loading threshold < 0.3); armswing, bradykinesia, ataxia, gait speed and steps loaded on factor one (eigenvalue of 2.4) which we interpreted as a gait disturbance sign; fine finger movement, dysdiadochokinesia speed and coordination on factor two (eigenvalue of 2.0) interpreted as a cerebellar sign;; tremor, cogwheeling and rigidity on factor three (eigenvalue of 1.8) interpreted as an extrapyramidal sign; pout and glabellar reflexes, fist palm side sequencing and reciprocal sequencing on factor four (eigenvalue of 1.8) interpreted as a frontal sign. We next tested the goodness-of-fit of the four-factor solution arising from the exploratory factor analysis in both individual countries on 70% of the remaining data. For this purpose, a model derived from the four-factor solution was derived and tested on 70% of the remaining data pooled over countries and at the individual site level using confirmatory factor analysis (see Table 3 and Fig. 1). Importantly, the variable vertical gaze was excluded from further analyses as it did not consistently load on any factor. A schematic illustration of the model used in our confirmatory factor analysis can be seen in Fig. 1.

**Table 3:** Confirmatory factor analysis

	NEUROEX items factor loadings	Cuba	Dominican Republic	Peru	Venezuela	Mexico	Across sites
Frontal signs	Pout reflex	0.3	0.5	0.3	0.3	0.5	0.4
	Glabellar reflex	0.3	0.3	0.3	0.3	0.5	0.4
	FPS sequencing	0.6	0.5	0.4	0.5	0.3	0.5
	Reciprocal sequencing	0.5	0.3	0.4	0.4	0.4	0.5
Extrapyramidal signs	Tremor	0.4	0.4	0.5	0.5	0.4	0.5
	Cogwheeling	0.5	0.3	0.6	0.4	0.4	0.5
	Rigidity	0.8	0.4	0.6	0.3	0.3	0.5
Cerebellar signs	Fine finger movement	0.7	0.6	0.7	0.6	0.5	0.6
	Dysdiadochokinesia speed	0.8	0.9	0.7	0.7	0.6	0.7
	Dysdiadochokinesia coordination	0.7	0.8	0.8	0.5	0.7	0.7
Gait disturbance signs	Armswing	0.4	0.6	0.7	0.6	0.6	0.6
	Gait –steps	0.5	0.6	0.3	0.4	0.4	0.3
	Gait –time	0.3	0.5	0.3	0.4	0.4	0.4
	Ataxia	0.7	0.5	0.9	0.7	0.8	0.7
	Bradykinesia	0.9	0.8	0.9	0.9	0.9	0.9
	Goodness of fit						
	χ^2^ (81)	531.0	480.5	418.0	323.6	291.0	1072.0
	TLI	0.91	0.90	0.95	0.95	0.93	0.94
	RMSEA	0.05	0.06	0.05	0.05	0.05	0.04
	AIC	58,147.3	40,061.3	51,287.9	35,137.0	41,058.3	216,584.0
	Measurement invariance model	Without constraints	With constraints	Difference			
	χ^2^	1942.7	3342.3	χ^2^ change		1399.7	
	Df	405	449	df change		44	
	TLI	0.92	0.89	*p*-value		*p* < 0.0001	
	RMSEA	0.05	0.07				
	AIC	200,452.1	289,429.0				

Confirmatory factor analysis with four-factor solution on 70% of the data, derived from the exploratory factor analysis on randomly selected 30% of the data. Goodness of fit parameters and loading coefficients by country, pooled over countries, and test of measurement invariance over countries. *AIC* Akaike’s Information Criterion, *FPS* fist palm side, *RSMA* Root mean square error of approximation, *TLI* Tucker-Lewis index

Across sites, a good fit was found for the variables derived from the NEUROEX assessment in our proposed four-factor model (χ^2^ = 1072.0.1; *p* < 0.001; df = 81; AIC = 216,584.0; TLI = 0.94; RMSEA = 0.04). Moderate to high factor loadings were found for all variables on the respective four factors (Table 3 and Fig. 1). Reasonable goodness-of-fit of the model was found as well for individual countries, with RMSEA varying between 0.05 and 0.06 with the highest value found in the Dominican Republic, while TLI varied between 0.91 and 0.95 with the highest value found in Peru and Venezuela and the lowest in the Dominican Republic. Overall moderate to high factor loadings were found across all countries. Measurement invariance analysis over all sites, revealed an acceptable fit of the constrained model (TLI = 0.89; RMSEA = 0.07).

### Poisson regression analyses

Poisson regression analyses were run to explore the association between 10/66 dementia at baseline and trichotomised factor scores respectively reflecting less-impaired, mildly-impaired and severely-impaired levels for the four groups of neurological signs (Table 4). Three models, one unadjusted, one adjusted for socio-demographical variables and one adjusted for socio-demographical variables and general indicators of health status were run separately for each group of neurological signs. In all models, factor scores of latent neurological signs were significantly associated with dementia at baseline. After full adjustment for confounders, the highest prevalence ratios were found for frontal (PR_heavily-impaired_ = 6.7 [5.0–8.9]), followed by extrapyramidal (PR_heavily-impaired_ = 3.3 [2.5–4.3]), cerebellar (PR_heavily-impaired_ = 2.9 [2.9–3.7]), and gait disturbance signs (PR_heavily-impaired_ = 2.0 [1.7–2.4]). Prevalence ratios were progressively increased in heavily-impaired subjects compared to mildly-impaired subjects.

**Table 4:** Poisson regression analysis

	Prevalence ratios (PR) with 95% CI		
	Unadjusted model	Adjusted model 1	Adjusted model 2
	PR	PR	PR
Frontal signs			
Mildly-impaired	4.7 (3.8–5.8)	3.9 (3.1–4.9)	3.8 (2.9–4.9)
Heavily-impaired	15.0 (12.1–18.6)	9.5 (7.4–12.1)	6.7 (5.0–8.9)
Extrapyramidal signs			
Mildly-impaired	4.2 (3.4–5.0)	2.9 (2.4–3.6)	2.5 (2.0–3.1)
Heavily-impaired	10.0 (8.1–12.3)	5.4 (4.3–6.9)	3.3 (2.5–4.3)
Cerebellar signs			
Mildly-impaired	4.2 (3.6–5.0)	2.8 (2.4–3.4)	2.4 (2.0–3.0)
Heavily-impaired	8.9 (7.2–10.7)	4.8 (3.9–6.0)	2.9 (2.2–3.7)
Gait disturbance signs			
Mildly-impaired	3.5 (2.3–5.1)	2.5 (1.7–3.7)	2.1 (1.3–3.3)
Heavily-impaired	3.9 (3.3–4.5)	2.3 (1.9–2.7)	2.0 (1.7–2.4)

Multivariate regression analysis of dementia at baseline by four factor scores derived from the NEUROEX assessment at baseline. Prevalence ratios (PR) in the sample pooled across countries (with 95% CI) are shown for an unadjusted model, a model adjusted for socio-demographical variables (adjusted for gender, educational level, food insecurity, income insecurity, number of assets and age; adjusted model 1) and a model adjusted for socio-demographical variables and general indicators of health status (adjusted for gender, educational level, food insecurity, income insecurity, number of assets, age, depression, care dependence, clinically diagnosed stroke, diabetes and hypertension; adjusted model 2)

### Cox proportional hazard regression analyses

Cox proportional hazard regression analyses were run to predict mortality at follow up from trichotomised factor scores, respectively reflecting less-impaired, mildly-impaired and severely-impaired levels for the four neurological signs (Table 5). As for the Poisson regression analysis, adjusted and unadjusted models were run. In all models, factor scores of all latent neurological signs significantly predicted mortality at follow up. Hazard-ratios were progressively increased in heavily-impaired subjects compared to mildly-impaired subjects. For the fully adjusted model, the highest-hazard ratios were found for frontal (HR_heavily-impaired_ = 1.6 [1.4–1.8), followed by extrapyramidal (HR_heavily-impaired_ = 1.4 [1.3–1.6]), and cerebellar signs (HR_heavily-impaired_ = 1.3 [1.1–1.5]). Gait disturbance signs predicted mortality in the unadjusted model (HR_heavily-impaired_ = 1.1 [1.0–1.2]), but not in the fully adjusted models (HR_heavily-impaired_ = 1.0 [0.9–1.0]). Sensitivity analyses were carried out using distinct data binning on the gait disturbance signs: a) based on the 90th percentile; b) by dividing the factor scores in five bins. The analysis based on the 90th percentile binarisation showed that gait disturbance predicted mortality at follow up in both the unadjusted (HR_impaired_ = 1.3 [1.2–1.4]) and the fully adjusted model (HR_impaired_ = 1.1 [1.1–1.3]). The second sensitivity analysis also revealed that higher gait disturbance predicted mortality at follow up both in the unadjusted (HR_heavily-impaired_ = 1.4 [1.2–1.5]; HR_severely-impaired_ = 1.3 [1.2–1.4]) and fully adjusted model (HR_heavily-impaired_ = 1.1 [1.0–1.2]; HR_severely-impaired_ = 1.1 [1.0–1.2]).

**Table 5:** Cox proportional hazard model regression analysis

	Hazard ratios (HR) with 95% CI		
	Unadjusted model	Adjusted model 1	Adjusted model 2
	HR	HR	HR
Frontal signs			
Mildly-impaired	1.2 (1.1–1.2)	1.1 (1.1–1.2)	1.1 (1.1–1.2)
Heavily-impaired	2.1 (2.0–2.3)	1.9 (1.7–2.1)	1.6 (1.4–1.8)
Extrapyramidal signs			
Mildly-impaired	1.3 (1.2–1.3)	1.2 (1.1–1.2)	1.1 (1.1–1.2)
Heavily-impaired	1.9 (1.7–2.1)	1.7 (1.5–1.9)	1.4 (1.3–1.6)
Cerebellar signs			
Mildly-impaired	1.5 (1.4–1.6)	1.4 (1.3–1.5)	1.3 (1.2–1.4)
Heavily-impaired	1.7 (1.5–1.9)	1.6 (1.4–1.7)	1.3 (1.1–1.5)
Gait disturbance signs			
Mildly-impaired	1.1 (1.0–1.3)	1.0 (0.9–1.2)	1.0 (0.8–1.1)
Heavily-impaired	1.1 (1.0–1.2)	1.1 (1.0–1.1)	1.0 (0.9–1.0)

Multivariate prediction analysis of mortality at follow up by four factor scores derived from the NEUROEX assessment at baseline. Hazard ratios (HR) pooled over countries (with 95% CI) are shown for an unadjusted model, a model adjusted for socio-demographical variables (adjusted for gender, educational level and age; adjusted model 1) and a model adjusted for socio-demographical variables and general indicators of health status (adjusted for gender, educational level, age, depression, clinically diagnosed stroke, diabetes, hypertension and dementia; adjusted model 2)

## Discussion

In this project, we aimed to investigate the construct and predictive validity of the NEUROEX battery assessing neurological symptoms among older adults in five Latin American countries. Dimensionality estimation of neurological symptoms from the NEUROEX assessment, revealed four groups of neurological signs which are in part anchored on vulnerability of distinct brain regions: frontal, extrapyramidal, cerebellar, and gait disturbance signs. Poisson and Cox regression models provided evidence for the predictive validity of all groups of signs on dementia and mortality.

### Prevalence of neurological symptoms among older adults in LMICs

The prevalence of neurological symptoms was in the range expected from previous works assessing the same question in high income countries [39]. However, variation in prevalence was observed for different sites (see Table 1). Distinct reasons could explain this finding: differences could be caused by diversity in the medical coverage and in the medical service found in the countries examined, as exemplified by Cuba’s health care system, which is considered one of the most effective in Latin America [40]. Moreover, the setting and the background of the community health worker could affect the variance found for the prevalence of neurological symptoms across sites (e.g.: training level of the community health worker; medical student versus practitioner).

### Exploratory factor analysis and confirmatory factor analysis

A four-factor principal component analysis on 30% randomly selected data consistently loaded neurological symptoms on four latent factors (see results and table in the Additional file 1 on exploratory factor analysis). A subsequent confirmatory factor analysis on the remaining 70% of the data confirmed suitability of four dimensions of neurological sings that can be associated with failure of distinct brain systems showing partial overlap with frontal, extrapyramidal, cerebellar, and gait disturbance signs [9–11]. For example, the pout reflex is a frontal release sign related to impaired inhibitory function of the frontal lobes, and is common in neurodegenerative diseases targeting the frontal lobes, such as frontotemporal lobar degeneration and vascular dementia [10]. Sequencing impairments and related executive dysfunctions are also related to damage of the frontal lobes [41], and are found in distinct dementia types ranging from Alzheimer’s disease, to frontotemporal and vascular dementia [41, 42]. Tremor, cogwheeling and rigidity are typical symptoms of extrapyramidal deficits of the striatopallidonigral system as encountered in Parkinson’s disease [13]. Difficulties in fine finger movements and dysdiadochinesia are well known impairments related to cerebellar dysfunction as found in multiple system atrophy or multiple sclerosis [12]. However, we advise caution in the interpretation of the findings as neurological symptoms are not exclusive to one specific neurological syndrome and may result from lesions in different brain areas. This inconsistency might result from different aetiologies underlying related neurological signs or by a more distributed failure of several brain structures involved in the manifestation of specific symptoms. For example, a positive glabellar reflex is believed to be caused by a lack of inhibitory function from the frontal lobes, but has been mainly associated with extrapyramidal diseases such as Parkinson’s disease [43]. Gait ataxia, commonly considered a cerebellar sign, can also be caused by frontal dysfunctions or disorders of the peripheral nervous system [13], but was associated with the gait disturbance sign in our study. Bradykinesia, which is considered an extrapyramidal sign, consistently loaded on the gait disturbance sign in our study, and may indicate a measurement artifact rather reflecting general slowing of the locomotor system than true bradykinesia. In particular, the latent variable gait disturbance sign might include mixed types of symptoms related to locomotion dysfunction with more complex diffuse contributions – also of non-neurological origin, e.g. arthritis, or impairments of the respiratory and vascular system among others.

### Link between neurological signs and dementia at baseline

All neurological signs had high prevalence ratios and were positively associated with dementia at baseline, even after adjustment for socio-demographics factors and general indicators of health status. These results are in line with previous research associating neurological symptoms such as primitive reflexes and parkinsonism with Alzheimer’s disease and other dementias [14, 15, 17]. Notably, in a prospective, longitudinal study of community-dwelling older people who did not have dementia or Parkinson disease at baseline, Louis and colleagues showed that baseline mild extrapyramidal signs can be used as a predictor of incident dementia [17]. In our study, frontal signs had the highest association with dementia, in line with both cross-sectional and longitudinal studies reporting deficits in frontal functioning which are associated with dementia in a variety of conditions ranging from vascular dementia, to Alzheimer’ disease, Parkinson’s disease dementia, and frontotemporal dementia [44–48] . Our findings support the notion that neurological signs, could be used as non-cognitive, education-independent, and culture-independent determinants of dementia. Importantly, our findings pave the way for a whole new line of research questions, which aim at investigating the predictive validity of neurological signs on longitudinal general health indicators and mental health disorders, including among others hypertension, alcohol abuse, head injury, major depression, Parkinson’s disease and distinct dementia subtypes such as Alzheimer’s disease, frontotemporal, and vascular dementia.

### Link between neurological signs and mortality at follow up

Cox hazard regression models revealed a positive association of neurological signs with mortality at follow up. These results remained statistically significant after adjustment and are in line with other longitudinal studies reporting a robust association of neurological symptoms such as parkinsonism and primitive reflexes with mortality at follow up [15, 16, 18]. In general, our findings are in line with the well-known association between frailty and mortality, particularly in advanced adulthood, suggesting that neurological symptoms might increase vulnerability of older adults and act as frailty and disability indicators increasing the risk of mortality [49, 50]. A level of uncertainty remains for gait disturbance signs, were only modest associations were found with mortality at follow up, as shown by the unadjusted model and the sensitivity analyses. Longitudinal findings from the Sidney Memory and Aging Study revealed a strong association of gait and motor abnormalities with dementia and mortality among older adults [51]. This difference might be caused by a suboptimal trichotomisation of the corresponding factor scores in our study, which may primarily result in the detection of individuals without gait impairments and or by the possibility that gait disturbance signs in our study might reflect symptoms with mixed aetiology rather than specific neurological dysfunctions.

### Limitations

Several limitations need to be addressed when interpreting our findings. Fully trained neurologists and psychiatrists assessed the participant in Cuba, medical students performed most of the assessment in Venezuela and Dominican Republic, while in Peru social workers and in Mexico General Practitioners were mainly involved. Although all the interviewers received the same training, we cannot exclude that interobserver variability could influence the differences in prevalence of neurological symptoms across countries. Findings cannot be generalised to higher income countries or to other LMICs, as the study was only conducted in a selected group of Latin American countries. High response rate was achieved by the use of catchment areas but with a general loss of generalisability, as the findings might not be applicable outside these areas and similar districts. Moreover, we did not explore differences between rural and urban areas. Although measurement of invariance across sites was acceptable, our results might be influenced by general methodological issues such as systematic differences in the way in which measures are being administered or coded, in the way in which participants are responding to interviews, and in misclassification of neurological symptoms and clinical diagnoses across sites.

## Conclusions

In conclusion, our results support construct and predictive validity of the NEUROEX assessment. Our findings relate neurological symptoms to groups of neurological signs, with dementia at baseline, and with mortality at follow up in older adults living in five Latin American countries. This study informs about the feasibility and utility of including a structured assessment of neurological signs as part of a survey of health and ageing in LMICs, and how this assessment can be used as a research tool to explore determinants of dementia and predictors of mortality [52, 53].

## Additional file

Additional file 1: Supplement. (DOCX 60 kb)

## Abbreviations

FPS: First palm side
LMICs: Low and middle-income countries
RMSEA: Root mean square error of approximation
TLI: Tucker–Lewis Index

## References

[1] Vos T, Barber RM, Bell B, Bertozzi-Villa A, Biryukov S, Bolliger I, Charlson F, Davis A, Degenhardt L, Dicker D (2015). Global, regional, and national incidence, prevalence, and years lived with disability for 301 acute and chronic diseases and injuries in 188 countries, 1990–2013: a systematic analysis for the global burden of disease study 2013. Lancet.

[2] Prince M, Patel V, Saxena S, Maj M, Maselko J, Phillips MR, Rahman A (2007). No health without mental health. Lancet.

[3] Organization WH: The world health report 2001: mental health: new understanding, new hope: World Health Organization; 2001.

[4] Prince M, Ali G-C, Guerchet M, Prina AM, Albanese E, Wu Y-T (2016). Recent global trends in the prevalence and incidence of dementia, and survival with dementia. Alzheimers Res Ther.

[5] Broe GA, Akhtar AJ, Andrews GR, Caird FI, Gilmore AJ, McLennan WJ (1976). Neurological disorders in the elderly at home. J Neurol Neurosurg Psychiatry.

[6] Broe GA, Jorm AF, Creasey H, Grayson D, Edelbrock D, Waite LM, Bennett H, Cullen JS, Casey B (1998). Impact of chronic systemic and neurological disorders on disability, depression and life satisfaction. Int J Geriatr Psychiatry.

[7] Jack CR, Knopman DS, Jagust WJ, Petersen RC, Weiner MW, Aisen PS, Shaw LM, Vemuri P, Wiste HJ, Weigand SD (2013). Tracking pathophysiological processes in Alzheimer's disease: an updated hypothetical model of dynamic biomarkers. The Lancet Neurology.

[8] Bateman RJ, Xiong C, Benzinger TL, Fagan AM, Goate A, Fox NC, Marcus DS, Cairns NJ, Xie X, Blazey TM (2012). Clinical and biomarker changes in dominantly inherited Alzheimer's disease. N Engl J Med.

[9] Larner (2011). A dictionary of neurological signs, second edition edn.

[10] Ropper A.H. SMA, Klein J.K.: Chapter 1. Approach to the patient with neurologic disease in: Adams and Victor's principles of neurology. 10 edn. McGraw-hill; 2014.

[11] Mesulam M: Chapter 1. Behavioral Neuroanatomy: large-scale networks, association cortex, frontal syndromes, the limbic system, and hemispheric specializations. In: Principles of Behavioral and Cognitive Neurology. Second edition edn. Oxford University Press; 2000.

[12] Daroff R. JJ, Mazziotta J., Pomeroy S.: Bradley's neurology in clinical practice, 7 edn: Elsevier; 2012.

[13] Fahn S JJ, Hallett M: Principles and practice of movement disorders: Elsevier; 2011.

[14] Girling DM, Berrios GE (1990). Extrapyramidal signs, primitive reflexes and frontal lobe function in senile dementia of the Alzheimer type. Br J Psychiatry.

[15] Burns A, Jacoby R, Levy R (1991). Neurological signs in Alzheimer's disease. Age Ageing.

[16] Bennett DA, Beckett LA, Murray AM, Shannon KM, Goetz CG, Pilgrim DM, Evans DA (1996). Prevalence of parkinsonian signs and associated mortality in a community population of older people. N Engl J Med.

[17] Louis ED, Tang MX, Mayeux R (2004). Parkinsonian signs in older people in a community-based study: risk of incident dementia. Arch Neurol.

[18] Wilson RS, Schneider JA, Beckett LA, Evans DA, Bennett DA (2002). Progression of gait disorder and rigidity and risk of death in older persons. Neurology.

[19] Bakchine S, Lacomblez L, Palisson E, Laurent M, Derouesne C (1989). Relationship between primitive reflexes, extra-pyramidal signs, reflective apraxia and severity of cognitive impairment in dementia of the Alzheimer type. Acta Neurol Scand.

[20] Prince M, Ferri CP, Acosta D, Albanese E, Arizaga R, Dewey M, Gavrilova SI, Guerra M, Huang Y, Jacob KS (2007). The protocols for the 10/66 dementia research group population-based research programme. BMC Public Health.

[21] Prina AM, Acosta D, Acostas I, Guerra M, Huang Y, Jotheeswaran AT, Jimenez-Velazquez IZ, Liu Z, Llibre Rodriguez JJ, Salas A (2017). Cohort profile: the 10/66 study. Int J Epidemiol.

[22] Prince M, Acosta D, Ferri CP, Guerra M, Huang Y, Llibre Rodriguez JJ, Salas A, Sosa AL, Williams JD, Dewey ME (2012). Dementia incidence and mortality in middle-income countries, and associations with indicators of cognitive reserve: a 10/66 dementia research group population-based cohort study. Lancet.

[23] Prince MJ, de Rodriguez JL, Noriega L, Lopez A, Acosta D, Albanese E, Arizaga R, Copeland JR, Dewey M, Ferri CP (2008). The 10/66 dementia research Group's fully operationalised DSM-IV dementia computerized diagnostic algorithm, compared with the 10/66 dementia algorithm and a clinician diagnosis: a population validation study. BMC Public Health.

[24] Prince M, Acosta D, Chiu H, Scazufca M, Varghese M, Dementia Research G (2003). Dementia diagnosis in developing countries: a cross-cultural validation study. Lancet.

[25] Llibre Rodriguez JJ, Ferri CP, Acosta D, Guerra M, Huang Y, Jacob KS, Krishnamoorthy ES, Salas A, Sosa AL, Acosta I (2008). Prevalence of dementia in Latin America, India, and China: a population-based cross-sectional survey. Lancet.

[26] Copeland JR, Dewey ME, Griffiths-Jones HM (1986). A computerized psychiatric diagnostic system and case nomenclature for elderly subjects: GMS and AGECAT. Psychol Med.

[27] Hall KS, Gao S, Emsley CL, Ogunniyi AO, Morgan O, Hendrie HC (2000). Community screening interview for dementia (CSI 'D'); performance in five disparate study sites. Int J Geriatr Psychiatry.

[28] Ganguli M, Chandra V, Gilby JE, Ratcliff G, Sharma SD, Pandav R, Seaberg EC, Belle S (1996). Cognitive test performance in a community-based nondemented elderly sample in rural India: the indo-U.S. cross-National Dementia Epidemiology Study. Int Psychogeriatr.

[29] Guerra M, Prina AM, Ferri CP, Acosta D, Gallardo S, Huang Y, Jacob KS, Jimenez-Velazquez IZ, Llibre Rodriguez JJ, Liu Z (2016). A comparative cross-cultural study of the prevalence of late life depression in low and middle income countries. J Affect Disord.

[30] Sousa RM, Ferri CP, Acosta D, Guerra M, Huang Y, Jacob K, Jotheeswaran A, Hernandez MA, Liu Z, Pichardo GR (2010). The contribution of chronic diseases to the prevalence of dependence among older people in Latin America, China and India: a 10/66 dementia research group population-based survey. BMC Geriatr.

[31] Joreskog KG. Latent variable modeling with ordinal variables. Stat Model Latent Variables. 1993:163–71.

[32] Joreskog KG (1994). Structural equation modeling with ordinal variables. Inst Math S.

[33] Castro-Costa E, Dewey M, Stewart R, Banerjee S, Huppert F, Mendonca-Lima C, Bula C, Reisches F, Wancata J, Ritchie K (2008). Ascertaining late-life depressive symptoms in Europe: an evaluation of the survey version of the EURO-D scale in 10 nations. The SHARE project. Int J Meth Psych Res.

[34] Anderson DR, Burnham KP, White GC (1998). Comparison of Akaike information criterion and consistent Akaike information criterion for model selection and statistical inference from capture-recapture studies. J Appl Stat.

[35] Tucker LR, Lewis C (1973). Reliability coefficient for maximum likelihood factor-analysis. Psychometrika.

[36] Browne: Alternative ways of assessing model fit. Sage Focus Editions 1993, 154:136–136.

[37] DiStefano (2009). Undertsanding and using factor scores: considerations for the applied researcher. Pract Assesment Res Eval.

[38] Jotheeswaran AT, Williams JD, Prince MJ (2010). Predictors of mortality among elderly people living in a south Indian urban community; a 10/66 dementia research group prospective population-based cohort study. BMC Public Health.

[39] Odenheimer G, Funkenstein HH, Beckett L, Chown M, Pilgrim D, Evans D, Albert M (1994). Comparison of neurologic changes in 'successfully aging' persons vs the total aging population. Arch Neurol.

[40] Cooper RS, Kennelly JF, Ordunez-Garcia P (2006). Health in Cuba. Int J Epidemiol.

[41] Rabinovici Gil D., Stephens Melanie L., Possin Katherine L. (2015). Executive Dysfunction. CONTINUUM: Lifelong Learning in Neurology.

[42] Viskontas IV, Possin KL, Miller BL (2007). Symptoms of frontotemporal dementia provide insights into orbitofrontal cortex function and social behavior. Ann N Y Acad Sci.

[43] Masdeu JC (2011). BJ: localization in clinical neurology, 6th edn: Wolters Kluwer health.

[44] Piccirilli M, D'Alessandro P, Finali G, Piccinin G (1997). Early frontal impairment as a predictor of dementia in Parkinson's disease. Neurology.

[45] Hogan DB, Ebly EM (1995). Primitive reflexes and dementia: results from the Canadian study of health and aging. Age Ageing.

[46] Vreeling FW, Houx PJ, Jolles J, Verhey FR (1995). Primitive reflexes in Alzheimer's disease and vascular dementia. J Geriatr Psychiatry Neurol.

[47] Murphy RR, Abner EL, Jicha GA (2015). Frontal release signs predict future decline in subjects with intact cognition and mild cognitive impairment. Alzheimers Dement.

[48] Harrington MG, Chiang J, Pogoda JM, Gomez M, Thomas K, Marion SD, Miller KJ, Siddarth P, Yi X, Zhou F (2013). Executive function changes before memory in preclinical Alzheimer's pathology: a prospective, cross-sectional, case control study. PLoS One.

[49] Llibre Jde J, Lopez AM, Valhuerdi A, Guerra M, Llibre-Guerra JJ, Sanchez YY, Bosch R, Zayas T, Moreno C (2014). Frailty, dependency and mortality predictors in a cohort of Cuban older adults, 2003-2011. MEDICC Rev.

[50] At J, Bryce R, Prina M, Acosta D, Ferri CP, Guerra M, Huang Y, Rodriguez JJ, Salas A, Sosa AL (2015). Frailty and the prediction of dependence and mortality in low- and middle-income countries: a 10/66 population-based cohort study. BMC Med.

[51] Waite LM, Grayson DA, Piguet O, Creasey H, Bennett HP, Broe GA (2005). Gait slowing as a predictor of incident dementia: 6-year longitudinal data from the Sydney older persons study. J Neurol Sci.

[52] Ravindranath V, Dang HM, Goya RG, Mansour H, Nimgaonkar VL, Russell VA, Xin Y (2015). Regional research priorities in brain and nervous system disorders. Nature.

[53] Baingana F (2015). al'Absi M, Becker AE, Pringle B: global research challenges and opportunities for mental health and substance-use disorders. Nature.

