# Supplementary material for: Neurological signs as early determinants of dementia and predictors of mortality among older adults in Latin America: a 10/66 study using the NEUROEX assessment
Source: BMC Neurol. 2018 Oct 3;18:163. doi: 10.1186/s12883-018-1167-4 (PMC6168999; doi:10.1186/s12883-018-1167-4)
Supplement: Supplementary file 1 — Supplement. (DOCX 60 kb) [file 12883_2018_1167_MOESM1_ESM.docx]

**Supplementary Material for**

“Neurological signs as early determinants of dementia and predictors of mortality among older adults in Latin America: a 10/66 study using the NEUROEX assessment.”

**by Lorenzo Pasquini¹ ², Jorge Llibre Guerra², Martin Prince¹, Kia-Chong Chua¹, A. Matthew Prina¹***

**Supplementary Methods: 1**

- Exploratory factor analysis

**Supplementary Results: 1**

- Exploratory factor analysis

**Supplementary References**

**Supplementary Tables: 1**

- Table S1: Exploratory factor analysis

**Appendix: 1**

- NEUROEX assessment

**Supplementary Methods**

**Exploratory factor analysis**

Exploratory factor analysis is a data driven reduction method used to reduce a data set from a group of interrelated variables to a smaller set of factors. Exploratory factor analysis was used to estimate dimensionality of the selected NEUROEX items into four factors. For this aim, a four-factor solution principal component analysis was performed on the selected NEUROEX items based on the polychoric correlation matrix using randomly selected 30% of the data across countries [1, 2]. Inspection of the item’s correlation matrix (Pearson’s values range between 0.3-0.8) and of linear relationships between the variables confirmed suitability of the data. Common techniques as the Bartlett’s test of sphericity, the Kaiser-Meyer-Olkin (KMO) measure of sampling adequacy, Kaiser’s criterion, scree tests and Horn’s parallel analysis [3] were used as additional criteria to estimate exploratory factor analysis reliability. The cut off used to assume that an item loaded on a given factor was 0.3. A varimax rotation was carried out and an eigenvalue of one was used as initial extraction criterion [4].

**Supplementary Results**

**Exploratory factor analysis**

Exploratory factor analysis was performed on testlets derived from the NEUROEX assessment through four-factor principal component analyses on randomly selected 30% of the data (Table S1). While a four-factor solution was not the only possible solution, Kaiser’s criterion, scree plots and Horn’s parallel analysis supported a four-factor solution. Bartlett’s test of sphericity was significant (p<0.05) and the KMO index were in the expected range (>0.6). All factors together explained 51.0% of the total variance. The loading structure of the pooled dataset classified vertical gaze as not consistently loading on any factor (factor loading threshold <0.3); armswing, bradykinesia, ataxia, gait speed and steps on factor one (eigenvalue of 2.4) which we interpreted as a gait disturbance sign; fine finger movement, dysdiadochokinesia speed and coordination on factor two (eigenvalue of 2.0) interpreted as a cerebellar sign; tremor, cogwheeling and rigidity on factor three (eigenvalue of 1.8) interpreted as an extrapyramidal sign; pout and glabellar reflexes, fist palm side sequencing and reciprocal sequencing on factor four (eigenvalue of 1.8) interpreted as a frontal sign.

**Supplementary References**

1. Joreskog KG: Latent Variable Modeling with Ordinal Variables. *Statistical Modelling and Latent Variables* 1993:163-171.

2. Joreskog KG: Structural equation modeling with ordinal variables. *Inst Math S* 1994, 24:297-310.

3. Field: Discovering statistics using SPSS, Third edition edn. Londond: SAGE publications; 2009.

4. Castro-Costa E, Dewey M, Stewart R, Banerjee S, Huppert F, Mendonca-Lima C, Bula C, Reisches F, Wancata J, Ritchie K *et al*: Ascertaining late-life depressive symptoms in Europe: an evaluation of the survey version of the EURO-D scale in 10 nations. The SHARE project. *Int J Meth Psych Res* 2008, 17(1):12-29.

**Supplementary Tables**

**Table S1. Exploratory factor analysis on testlets derived from the NEUROEX assessment using randomly selected 30% of the dataset across countries. Loading threshold was 0.3 (in bold). FPS = fist palm side.**

| Pooled over sites | Loading coefficients | | | |
| --- | --- | --- | --- | --- |
| Items | Factor 1 | Factor 2 | Factor 3 | Factor 4 |
| Vertical gaze | 0.2 | 0.0 | 0.0 | 0.2 |
| Armswing | **0.6** | 0.0 | 0.2 | **0.3** |
| Gait – steps | **0.6** | 0.2 | 0.0 | **-0.3** |
| Gait – time | **0.7** | 0.2 | 0.0 | **-0.3** |
| Ataxia | **0.6** | 0.1 | 0.3 | 0.2 |
| Bradykinesia | **0.7** | 0.1 | 0.2 | 0.2 |
| Fine finger movement | 0.2 | **0.6** | 0.2 | 0.2 |
| Dysdiadochokinesia speed | 0.1 | **0.8** | 0.1 | 0.0 |
| Dysdiadochokinesia coordination | 0.1 | **0.7** | 0.1 | 0.1 |
| Glabellar reflex | 0.1 | 0.2 | 0.0 | **0.4** |
| Pout reflex | 0.1 | 0.0 | 0.1 | **0.6** |
| FPS sequencing | 0.1 | **0.3** | 0.0 | **0.6** |
| Reciprocal sequencing | 0.1 | **0.4** | 0.0 | **0.5** |
| Tremor | 0.1 | 0.1 | **0.5** | **0.3** |
| Cogwheeling | 0.0 | 0.1 | **0.9** | 0.0 |
| Rigidity | 0.1 | 0.2 | **0.8** | 0.0 |
| Eigenvalue | 2.4 | 2.0 | 1.8 | 1.8 |
| Explained variance in % | 15.6 | 12.7 | 11.4 | 11.3 |

**Appendix:**

**NEUROEX assessment**

**10/66 Dementia Research Group**

**Follow-up Incidence Study**

**April 2007**

**PHYSICAL AND NEUROLOGICAL EXAMINATION**

FOR THIS EXAMINATION, YOU WILL NEED:

STETHOSCOPE

SPHYGNOMANOMETER

FLEXIBLE CLOTH OR PAPER TAPE MEASURE (2 METRES LONG)

A PIECE OF STRING, 5 METRES LONG

A SET OF ELECTRONIC WEIGHING SCALES, AS PROVIDED FROM LONDON

---------------

IDENTIFICATION

---------------

Date of interview: DATE

dd/mm/yy

Enter date as day/month/year, e.g. 05/10/2003

Interviewer ID number: INTERID ##

Household ID number: HOUSEID #####

Participant ID number: PARTICID #

1. PULSE RATE

Count the pulse rate for 30 seconds at the wrist, and multiply by two to calculate

the rate per minute

PULSE RATE PER MINUTE pulse ###

2. BLOOD PRESSURE

2.1 SITTING BLOOD PRESSURE 1

With the participant sitting down and relaxed measure the blood pressure in the right arm.

Record the systolic and diastolic blood pressures.

SYSTOLIC BLOOD PRESSURE 1 bpsys1 ###

DIASTOLIC BLOOD PRESSURE 1 bpdias1 ###

2.2 SITTING BLOOD PRESSURE 2

Repeat the measurements and record the systolic and diastolic blood pressures.

SYSTOLIC BLOOD PRESSURE 2 bpsys2 ###

DIASTOLIC BLOOD PRESSURE 2 bpdias2 ###

2.3 STANDING BLOOD PRESSURE

Ask the participant to stand up, and record the systolic and diastolic blood pressures while standing up

SYSTOLIC BLOOD PRESSURE STANDING bpsys3 ###

DIASTOLIC BLOOD PRESSURE STANDING bpdias3 ###

2.4 ARM CIRCUMFERENCE

Measure the participant’s right upper arm circumference, with the arm relaxed.

Place the tape measure around the upper arm, and moved it up and down to locate

the thickest part (the largest circumference).

ARM CIRCUMFERENCE (CENTIMETRES) armcirc ##

3. CALF CIRCUMFERENCE

Ask the person to sit in a chair. It is important that the foot is resting on the floor,

and that the knee and ankle are each bent to a 90-degree angle. Place the tape measure around the calf,

and moved it up and down to locate the thickest part of the calf (the largest circumference).

CALF CIRCUMFERENCE (CENTIMETRES) calfcirc ##

4. SKULL CIRCUMFERENCE

Measure the participant’s skull circumference. Pass the tape measure just above the participant’s

eyebrows and round to the occipital pole (nuchal tuberosity) at the back of the skull.

This is the bony promontory where the neck strap muscles attach to the base of the skull.

SKULL CIRCUMFERENCE (CENTIMETRES) skcirc ##

NOW ASK THE PARTICIPANT TO STAND UP

5. WAIST CIRCUMFERENCE

Measure the participant’s waist circumference.

In women this is the narrowest part of the body between chest and hips.

In men this should be measured at the level of the umbilicus (belly button)

WAIST CIRCUMFERENCE (CENTIMETRES) wstcirc ###

6. HIP CIRCUMFERENCE

Measure the participant’s hip circumference.

Place the tape measure at the level of the greater trochanters (hip bones),

and include the maximum fullness of the buttocks

HIP CIRCUMFERENCE (CENTIMETRES) hipcirc ###

7. HEIGHT

Record the participant’s height, standing them up against a wall and

marking off their height using a book or similar resting on top of their head.

Then measure their height using the tape measure.

HEIGHT (CENTIMETRES) height ###

7.1 LEG LENGTH

Record the length of the participant’s leg from the iliac crest (pelvic bone)

down to the lateral malleoulus (ankle bone).

LEG LENGTH (CENTIMETRES) lglength ###

8. WEIGHT

Make sure the scales are placed on an even surface.

There is no need to ask the participant to strip, but you should ask them to remove any heavy outer garments

(overcoats/ jackets) and shoes. Body weight is measured to the nearest 0.1 kg.

WEIGHT (KILOGRAMS) weight ###.#

9 USE AND QUALITY OF DENTURES

9.1 Do you have dentures?

0 No

1 Yes denture #

IF NO, SKIP TO 10

9.2 OBSERVATION: ARE THE DENTURES WORN AT THE TIME OF EXAMINATION?

0 No

1 Yes dentex #

If yes, ask participant to remove dentures, before counting number of teeth

10. NUMBER OF TEETH (OWN TEETH)

10.1 UPPER JAW teeth1 ##

10.2 LOWER JAW teeth2 ##

10.3 Do you have problems with chewing?

0 No problems

1 Some problems

2 Many problems chewing #

11. LEVEL OF CONSCIOUSNESS

0 Alert

1 Not alert, but arousable by minor stimulation

2 Not alert, requires repeated stimulation to attend,

or requires strong or painful stimulation to make

movements

3 Responds only with reflex motor or automatic

effects, or totally unresponsive. lvlcons1 #

12. LEVEL OF CONSCIOUSNESS

How old are you?

What is the current month?

0 Answers both correctly

1 Answers one correctly

2 Both incorrect lvlcons2 #

13. LEVEL OF CONCIOUSNESS

Please close your eyes. Now open them.

Please make a fist, like this. Now let go.

0 Performs both tasks correctly

1 Performs one task correctly

2 Performs neither task correctly lvlcons3 #

14. BEST GAZE

Please follow my finger with your eyes, keeping your head still

TEST HORIZONTAL GAZE ONLY. MOVE FINGER FROM LEFT TO RIGHT TO THE FULL LIMIT OF GAZE.

IF EYES ARE PERMANENTLY DEVIATED TO ONE SIDE, THEN MOVE THE PARTICIPANT’S HEAD IN THAT DIRECTION

HAVING PLACED YOUR FACE IN THEIR LINE OF VISION. IF THE EYES REMAIN DEVIATED, CODE 2 FOR ‘FORCED DEVIATION’

0 Normal

1 Partial gaze palsy (gaze is abnormal in one or both eyes,

but forced deviation or total gaze paresis is not present)

2 Forced deviation or total gaze paresis is not overcome by

the oculocephalic manoeuvre bestgaze #

15. VISUAL

Tell me when you can see my finger wagging. Say yes, each time.

STAND DIRECTLY IN FRONT OF THE PARTICIPANT AND ASK THEM TO COVER THEIR RIGHT EYE.

CLOSE YOUR LEFT EYE AND ASK THEM TO LOOK AT YOUR RIGHT EYE.

MOVE YOUR WAGGING FINGER IN TOWARDS THE CENTRE FROM ALL FOUR QUADRANTS,

COMPARING YOUR VISUAL FIELD WITH THEIRS. (Introduce visual stimulus to patient’s visual field quadrants)

0 No visual loss

1 Partial hemianopia (some restriction to the left,

or right visual field but not to the midline)

2 Complete hemianopia (the whole of the left, or

right visual field is missing, to the midline)

3 Bilateral hemianopia (left and right field restriction visual) #

16. FACIAL PALSY

Please show me your teeth.

Please raise your eyebrows.

Please squeeze your eyes tight shut.

ON THE LAST COMMAND SEE IF IT IS POSSIBLE TO OPEN THE EYE BY PUSHING UPWARDS GENTLY ON THE EYEBROW

0 Normal symmetrical movements

1 Minor paralysis (flattened nasolabial fold, asymmetry on smiling)

2 Partial paralysis (total or near-total paralysis of lower face)

3 Complete paralysis of one or both sides

(absence of facial movement in the upper and lower face) fpalsy #

17. MOTOR ARM LEFT

Ask the participant to raise their left arm out in front of them, level with their shoulder,

palm facing upwards. Imitate for them what you would like them to do.

Say 'please hold your arm out in front of you like this.

Now please close your eyes, but hold your arms steady'

Observe for signs 'drifting'. Typically, if an arm has been affected by a stroke,

it will drift towards the midline and the palm will rotate to towards the downwards facing position.

If so, repeat the test to confirm the finding. Do not code if you feel fatigue or arthritis may have explained the drift.

If the participant cannot raise their arm to 90 degrees, then put the arm in this position for them,

and code 2 or 3 depending on whether they can make an effort to resist gravity.

If the participant cannot move their arm at all, score 4

(Elevate extremity to 90 degrees and score drift/movement)

No score for amputation and joint fusion

0 No drift

1 Drift

2 Some effort to resist gravity, but arm falls

3 No effort against gravity, arm falls straight down

4 No movement motor1 #

18. MOTOR ARM RIGHT

Repeat instructions and rating as for left arm

0 No drift

1 Drift

2 Some effort to resist gravity, but arm falls

3 No effort against gravity, arm falls straight down

4 No movement motor2 #

19. MOTOR LEG LEFT

Ask the participant to lie flat on a bed or couch and then raise their left leg straight up to 30 degrees.

Say 'Now please close your eyes, but hold your leg steady'

Observe for 10 seconds for signs of 'drifting'.

If present, repeat the test to confirm the finding.

Do not code if you feel fatigue or arthritis may have explained the drift.

If the participant cannot raise their leg to 30 degrees, then put the leg in this position for them,

and code 2 or 3 depending on whether they can make an effort to resist gravity.

If the participant cannot move their arm at all, score 4

(No score for amputation and joint fusion)

0 No drift

1 Drift

2 Some effort to resist gravity, but leg falls

3 No effort against gravity, leg falls straight down

4 No movement motor3 #

20. MOTOR LEG RIGHT

Repeat instructions and rating as for left arm

0 No drift

1 Drift

2 Some effort to resist gravity, but leg falls

3 No effort against gravity, leg falls straight down

4 No movement motor4 #

21. LIMB ATAXIA

Please place your right heel on your left knee and run it carefully down your shin bone.

(POINT TO THE RELEVANT HEEL, KNEE AND SHIN AS YOU SAY THIS)

Now, please place your left heel on your right knee and run it carefully down your shin bone.

(POINT TO THE RELEVANT HEEL, KNEE AND SHIN AS YOU SAY THIS)

Now please come and sit down again in the chair

When the participant is seated, hold your index finger in front of them and say

‘Now please touch my finger, and touch your nose’. When they have done this, say ‘and touch my finger again,

and your nose, my finger, your nose’ etc.) MOVE YOUR FINGER A LITTLE TO MAKE THE TASK MORE DIFFICULT

(Score 0 if not tested deal to weakness)

0 Absence

1 Ataxia present in one limb (arm or leg)

2 Ataxia present in two limbs

(arm and leg on the same side) limbatx #

22. SENSORY

(Light touch to face, arm, trunk, and leg – compare side to side)

0 Normal, no sensory loss

1 Mild-to-moderate sensory loss (sensation is duller on the affected side,

but participant is aware of being touched)

2 Severe or total sensory loss (participant is not aware of being touched

in the face, arm, and leg) sensory #

23. BEST LANGUAGE

Show the participant the picture and say

‘Please tell me what is going on in this picture. Describe everything you see’

Show the participant the drawing of 5 objects and point to each in turn saying

‘What is this called, and this etc’?

Show the participant the 6 sentences and say

‘Please read this sentence, and this etc’

0 No aphasia

1 Mild to moderate aphasia, some obvious loss of fluency or comprehension but

can relate story from card so it can be understood by the interviewer

2 Severe aphasia, fragmentary speech only, story cannot be understood;

great need for inference, questioning and guessing by the interviewer

3 Mute, no usable speech

or auditory comprehension language #

24. DYSARTHRIA

(Evaluate speech clarity by patient repeating listed words)

0 Normal

1 Mild to moderate dysarthria

(patient slurs some words and can be understood with some difficulty)

2 Severe dysarthria (patient’s speech is so slurred as to be unintelligible

in the absence of or out of proportion

to any dysphasia, or is mute) dysarth #

25. EXTICTION AND INATTENTION

Ask the participant to put their hands on their knees.

Then touch their left hand and say ‘when I touch your left hand I want you to say “left”.

Then touch their right hand and say ‘when I touch your right hand I want you to say “right”.

Then touch both hands simultaneously and say, when I touch both hands at once I want you to say “both”.

Ask the participant to close their eyes, and test for sensory neglect by touching e.g. L/ R/ L/ R/ both

0 No abnormality

1 Does not notice stimulation to one side,

when both sides stimulated simultaneously

2 Profound inattention to one half of the body,

e.g does not recognize own hand or orients

to only one side of space

extinat #

25a.INSTRUCTIONS FOR INTERVIEWER: ADD THE SCORES FOR QUESTION 11 TO 25

NIHTOTAL ##

IF TOTAL SCORE IS 4 OR MORE, THIS PARTICIPANT SHOULD BE SELECTED FOR THE SECOND PHASE STROKE ASSESSMENT. REMEMBER TO TELL YOUR STUDY COORDINATOR.

26. LURIA'S TESTS FOR FRONTAL LOBE FUNCTION:

For the next test ask the participant to use their dominant hand,

unless prevented from doing so for example by stroke, amputation or arthritis.

Code below which hand has been used

26.1 WHICH HAND USED FOR PALM-FIST-SIDE?

1 dominant

2 non-dominant

WHICH HAND hand #

26.2 FIST-PALM-SIDE

Show a fist resting on your thigh while seated on a chair, next open the palm,

next turn the open hand to the side so that your little finger rests on your thigh.

26.2.1 LEARNING - FIST-PALM-SIDE

SAY: "I want you to watch my hand movements and then repeat them after I have finished". (ONE PER SECOND)

0 Requires only one demonstration

1 Requires 2-3 demonstrations

2 Requires 4-5 demonstrations

3 Unable to learn correctly within 5 demonstrations

(if so, do not attempt sequencing [below] and score 3 for neo8b)

LEARNING, DOMINANT HAND neo8a #

26.2.2 SEQUENCING - FIST-PALM-SIDE

SAY:" I now want you to repeat these movements five times’.

If more than one mistake is made then redemonstrate how it should be done and ask

the participant to try again

0 5 sequences correct

1 5 sequences performed with one mistake

2 5 sequences after one re-demonstration

3 Unable to complete 5 sequences correctly (include those who scored 3 on learning above)

SEQUENCING, DOMINANT HAND neo8b #

27. GAIT

Lay a five-metre length of string along the ground and ask the participant to walk

normally to the end of the string, turn round and walk back again observing:

27.1 NUMBER OF STEPS neo12a ##

MV=99

27.2 TIME (SECONDS) neo12b ###

MV=999

27.3 ARM SWING, RIGHT

0 normal arm swing

1 reduced arm swing

2 no arm swing

ARM SWING, RIGHT neo12c #

27.3.1 ARM SWING, LEFT

0 normal arm swing

1 reduced arm swing

2 no arm swing

ARM SWING, LEFT neo13d #

27.4 ATAXIA

0 normal gait

1 unsteady, broad-based gait

2 very unsteady broad-based gait

ATAXIA neo14e #

27.5 BRADYKINESIA

0 all movements normal speed

1 somewhat slow movements

2 very slow movements

BRADYKINESIA neo14g #

28. BED BOUND

INTERVIEWER: USING YOUR JUDGMENT FROM EVERYTHING YOU HAVE SEEN AND HEARD IN THE INTERVIEW, DESCRIBE THE PARTICIPANT'S INDEPENDENT LEVEL OF MOBILITY (THAT IS, WHAT THEY CAN MANAGE WITHOUT RECEIVING HELP). CODE THE HIGHEST NUMBER THAT APPLIES.

1. BED BOUND

2. CHAIR BOUND (CAN TRANSFER FROM BED TO CHAIR ONLY)

3. HOUSE BOUND (LIMITED MOBILITY AROUND THE HOUSE, BUT CAN NOT GO OUTSIDE ON THEIR OWN)

4. LIMITED MOBILITY OUTSIDE OF THE HOME

5. FREELY MOBILE OUTSIDE OF THE HOME, NO SIGNIFICANT RESTRICTIONS

bedb #
